# Prognostic Value of Sudomotor Small‐Fiber Function and Large Nerves for Accelerated Renal Decline in Type 2 Diabetes: A Real‐World Cohort Study

**DOI:** 10.1111/1753-0407.70286

**Published:** 2026-10-19

**Authors:** Qingyun Cai, Xiuqi Qiao, Sijia Fei, Yanxiang Luo, Lixin Guo, Qi Pan

**Affiliations:** ^1^ Department of Endocrinology, Beijing Hospital National Center for Gerontology, National Clinical Research Center for Gerontology, The Key Laboratory of Geriatrics of NHC, Institute of Geriatric Medicine, Chinese Academy of Medical Sciences Beijing China; ^2^ Chinese Academy of Medical Sciences and Peking Union Medical College Beijing China

**Keywords:** diabetic kidney disease, diabetic peripheral neuropathy, large nerve fiber, sudomotor function, type 2 diabetes

## Abstract

**Aims:**

To evaluate the longitudinal prognostic value of small‐ and large‐fiber peripheral nerve assessments for accelerated renal decline in patients with Type 2 diabetes (T2D).

**Methods:**

An initial cross‐sectional analysis evaluated concurrent neuro‐renal associations, while a subsequent longitudinal cohort assessed accelerated renal progression (eGFR loss > 3 mL/min/1.73 m^2^/year) using paired baseline and follow‐up data. Prognostic predictors were selected via LASSO regression. Model utility was assessed using area under the curve (AUC), net reclassification improvement (NRI), and decision curve analysis (DCA).

**Results:**

Cross‐sectional sudomotor small‐fiber dysfunction accurately mirrored concurrent renal impairment (*β* = 0.18, 95% CI: 0.10–0.26, *p* < 0.001). Longitudinally, ESCF demonstrated no independent prognostic value for accelerated renal decline, and its apparent longitudinal association with future eGFR was completely attenuated after adjusting for baseline renal function. LASSO regression retained right peroneal motor amplitude, serum albumin, platelet count, and SGLT2i use as robust prognostic factors. Notably, subgroup analyses revealed this large‐nerve predictive capacity was significant primarily in patients with established DKD (*p* for interaction = 0.006). Incorporating this neurofunctional phenotype into standard clinical models yielded a modest AUC improvement but achieved a clinically relevant continuous NRI of 30.35%, successfully recalibrating risk profiles. DCA confirmed a superior net clinical benefit.

**Conclusion:**

A distinct prognostic dissociation exists within the diabetic neuro‐renal axis. Sudomotor small‐fiber dysfunction primarily reflects established microvascular impairment, whereas large motor nerve degeneration demonstrates independent prognostic value for future accelerated renal decline. Incorporating this neurofunctional phenotype into traditional clinical models yields substantial individualized risk reclassification, thereby potentially aiding in identifying high‐risk individuals.

## Highlights

- A distinct prognostic dissociation exists within the diabetic neuro‐renal axis.
- Sudomotor small‐fiber dysfunction merely mirrors baseline microvascular impairment, whereas large motor nerve degeneration is independently associated with future accelerated renal decline.
- Incorporating this large‐nerve phenotype into traditional clinical models yields substantial individualized risk reclassification, improving overall prognostic stratification.

## Graphical abstract

A prognostic dissociation exists within the diabetic neuro‐renal axis: while small‐fiber dysfunction merely mirrors baseline damage, large motor nerve dysfunction is independently associated with accelerated renal decline. Integrating this macro‐structural phenotype into traditional models significantly improves risk reclassification, potentially aiding in the identification of hidden high‐risk patients.

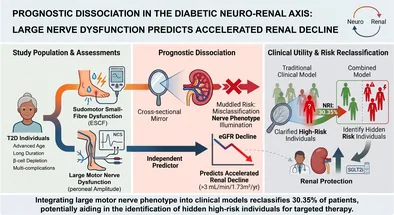

## 1 Introduction

Diabetic kidney disease (DKD) is the leading cause of end‐stage renal disease (ESRD) globally [1]. Advanced age, long duration of diabetes, poor glycemic control, depleted residual islet function, and multiple established micro‐ and macrovascular complications are well‐documented risk factors for accelerated renal function decline in patients with DKD [2]. However, traditional metabolic parameters (e.g., glycated hemoglobin and lipid profiles) have limited prognostic value in such high‐risk populations [3]. Established structural microvascular damage—rather than active metabolic fluctuations—is the primary driver of disease progression in patients with advanced diabetes [4]. Consequently, accurate risk stratification necessitates alternative prognostic approaches.

The peripheral nervous system and renal microvasculature share intrinsically linked ischemic and metabolic injury pathways, including hyperglycemia‐induced oxidative stress, accumulation of advanced glycation end‐products, and microvascular ischemia [5]. Therefore, peripheral nerve assessment presents a highly promising biomarker capable of directly reflecting structural end‐organ damage. SUDOSCAN reflects damage to postganglionic sympathetic C‐fibers, which is one of the earliest detectable complications and a sensitive indicator of systemic microvascular impairment in Type 2 diabetes [6]. Specifically, integrated diagnostic algorithms, such as the Sudoscan‐DKD risk score, demonstrate close cross‐sectional correlations with the baseline estimated glomerular filtration rate (eGFR) and albuminuria, exhibiting potential clinical utility in identifying patients at high risk of nephropathy [7]. However, longitudinal data remain scarce, with limited prospective studies yielding conflicting results. It remains unclear whether early functional small‐fiber impairment provides independent prognostic value for accelerated renal decline after adjusting for baseline eGFR and traditional risk factors, or merely acts as an epiphenomenon reflecting concurrent baseline microvascular damage. Clarifying this mechanism will be essential to determine the true longitudinal prognostic value of small‐fiber assessment.

To address this knowledge gap, we conducted a real‐world cohort study focusing on patients with advanced age, long disease duration, depleted residual islet function, and multiple established complications. To comprehensively map the neurofunctional spectrum, standard nerve conduction studies (NCS) were performed concurrently with ESCF assessments at baseline. The primary objective of this study was to determine the independent prognostic value of sudomotor small‐fiber nerve degeneration versus functional large‐fiber impairment in predicting accelerated renal decline.

## 2 Methods

### 2.1 Study Design and Participants

This real‐world, retrospective cohort study included adult patients with Type 2 diabetes who were admitted to Beijing Hospital between January 2020 and September 2025. Patients were systematically excluded based on the following predefined criteria: (1) incomplete baseline sudomotor small‐fiber assessment (Sudoscan), NCS, or routine laboratory data; (2) peripheral neuropathy of nondiabetic etiologies (e.g., vitamin B12 deficiency or neurotoxic drug exposure); (3) anatomical or physiological conditions precluding valid neurological testing, such as severe lower extremity arterial disease or limb amputation; (4) documented episodes of acute kidney injury at baseline or during the follow‐up period; and (5) active malignancy or a history of solid organ transplantation.

Following these exclusions, 694 eligible patients were enrolled in the baseline cross‐sectional analysis. Patients lacking follow‐up records were excluded from the subsequent longitudinal phase, resulting in a final longitudinal sub‐cohort of 234 patients with paired renal function data available at baseline and the final follow‐up endpoint. The median follow‐up duration for this longitudinal sub‐cohort was 2 years. The study was approved by the Institutional Review Board of Beijing Hospital (Approval Number 2024BJYYEC‐KY083‐02). A waiver of informed consent was granted due to the retrospective and anonymized nature of the clinical data.

### 2.2 Clinical and Biochemical Measurements

During the study period, baseline demographic and clinical data were extracted from electronic medical records during the participants' initial hospital admission, while longitudinal data were obtained at the final follow‐up endpoint.

Comprehensive clinical information included age, sex, duration of diabetes, and lifestyle factors (current smoking status and alcohol consumption). Detailed medical histories of patients with diabetes‐related complications (e.g., hypertension, diabetic retinopathy, and cardiovascular disease) and concurrent pharmacological treatments were recorded. Anthropometric measurements, including height, weight, and waist circumference, were obtained using standardized protocols. The body mass index (BMI) was calculated as weight in kilograms divided by the square of height in meters.

Venous blood and urine samples were collected uniformly after an overnight fast of at least 8 h. Biochemical analyses were performed using automated analyzers at the hospital's central laboratory. The laboratory panel included fasting blood glucose, glycated hemoglobin (HbA1c), fasting C‐peptide, lipid profiles, complete blood counts (including platelet and lymphocyte counts), and liver function tests (including serum albumin). The urinary albumin‐to‐creatinine ratio (UACR) was also assessed. The baseline and follow‐up eGFR were calculated using the Chronic Kidney Disease Epidemiology Collaboration equation [8].

### 2.3 Neurofunctional Evaluation

#### 2.3.1 Sudomotor Small‐Fiber Assessment Fibers

Sudomotor small‐fiber integrity was evaluated by measuring the electrochemical skin conductance of feet (ESCF) using the Sudoscan device (Impeto Medical, Paris, France). This noninvasive modality specifically assesses the function of postganglionic sympathetic cholinergic C‐fibers that innervate the sweat glands. During the assessment, the participants placed their bare hands and feet on stainless‐steel sensor plates for 2 min while a low‐voltage direct current (< 4 V) was applied. The mean ESCF, expressed in microsiemens (μS), was recorded. In this study, the baseline ESCF served as the primary quantitative surrogate for systemic microvascular small‐fiber impairment.

#### 2.3.2 Large‐Fiber Electrophysiological Assessment

The structural and functional integrity of large‐fibers was evaluated using standard NCS. Experienced neurophysiologists performed electrophysiological recordings using a Nicolet VikingQuest electromyography system (Natus Medical/Nicolet, Middleton, WI, USA). To ensure test reliability, extremity skin temperatures were rigorously maintained at ≥ 32°C throughout the procedure.

Although comprehensive evaluations were conducted to rule out nondiabetic etiologies, our quantitative longitudinal tracking specifically targeted the right lower extremity, designed to capture the progressive deterioration of the longest and most metabolically vulnerable axonal pathways. The evaluated parameters included motor and sensory conduction velocities, distal motor latencies, compound muscle action potential (CMAP) amplitudes, and sensory nerve action potential (SNAP) amplitudes.

### 2.4 Definition of Accelerated Renal Function Decline

The primary outcome of the longitudinal analysis was accelerated renal function decline. The annualized rate of eGFR change was calculated using a precise two‐point method. The absolute decline in renal function was determined by subtracting the eGFR at the final follow‐up endpoint from the baseline eGFR, which was subsequently divided by the exact individual follow‐up duration (expressed in years).

Guided by established prognostic literature and statistical feasibility considerations, the primary endpoint for accelerated renal decline was defined as an annualized eGFR loss of > 3 mL/min/1.73 m^2^/year [9, 10]. Clinically, this threshold represents a trajectory nearly five times faster than the physiological aging rate [11], allowing for early identification of active deterioration. Statistically, it yielded 79 incident events, which optimally maintained robust event‐per‐variable ratios for multivariable modeling. Furthermore, to ensure rigorous alignment with international guidelines, the stricter KDIGO‐recommended threshold of a rapid decline > 5 mL/min/1.73 m^2^/year was concurrently evaluated as a pre‐specified sensitivity analysis [12].

### 2.5 Statistical Analysis

Baseline continuous variables are presented as medians with interquartile ranges (IQRs) due to nonnormal distributions, and categorical variables are presented as frequencies and percentages. Differences across the three ESCF tertiles (T1, T2, and T3) were compared using the Kruskal–Wallis test for continuous variables and Pearson's chi‐square test for categorical variables.

Statistical analysis was conducted in sequential phases to comprehensively evaluate the neuro‐renal axis.

#### 2.5.1 Cross‐Sectional Analysis

Multivariable linear regression models were used to assess the cross‐sectional association between ESCF and baseline eGFR. To confirm independent correlations, sequential adjustments were made for demographic variables, traditional metabolic risk factors, and large‐fiber amplitude.

#### 2.5.2 Variable Selection and Longitudinal Prediction

Because the final longitudinal cohort (*n* = 234) was strictly defined by the availability of comprehensive baseline assessments and follow‐up data, no missing values were present for the primary clinical and neurofunctional covariates. To mitigate multicollinearity among the high‐dimensional baseline clinical variables (e.g., HbA1c, diabetes duration, and complications) and prevent overfitting, the least absolute shrinkage and selection operator (LASSO) logistic regression with 10‐fold cross‐validation was used to identify the most robust predictors of accelerated renal decline. The optimal penalty parameter (*λ*) was determined based on the minimum binomial deviance criteria (*λ*_min_). Notably, with 79 recorded primary outcome events in the cohort, our core LASSO‐derived model (incorporating six variables) yielded an event‐per‐variable (EPV) ratio of 13.1, which robustly satisfies the recommended epidemiological threshold of > 10 to minimize baseline overfitting.

Baseline ESCF and baseline eGFR were designated a priori as the core neurofunctional exposure and the baseline anatomical anchor, respectively. Consequently, they were deliberately exempted from the LASSO penalization process and deterministically forced into the final models. Subsequently, multivariable logistic regression models—including a LASSO‐derived model (incorporating the LASSO‐selected covariates alongside the pre‐specified ESCF and baseline eGFR) and a fully adjusted model—were constructed to evaluate the independent predictive value of small‐ and large‐fiber phenotypes for the primary outcome (> 3 mL/min/1.73 m^2^/year). Results are expressed as odds ratios (ORs) with 95% confidence intervals (CIs). This analysis was identically repeated for the sensitivity outcome (> 5 mL/min/1.73 m^2^/year).

#### 2.5.3 Evaluation of Sudomotor Predictive Attenuation

To evaluate whether the apparent longitudinal association between baseline ESCF and follow‐up renal function was confounded by baseline renal status, sequential multivariable linear regression models were utilized. Follow‐up eGFR was set as the dependent variable. We first assessed the association adjusting for demographic and traditional metabolic risk factors. Subsequently, baseline eGFR was added to the fully adjusted model to determine if the predictive value of ESCF would be attenuated, thereby assessing whether its prognostic capacity was independent of the baseline renal foundation.

#### 2.5.4 Subgroup and Interaction Analyses

To evaluate the robustness of peroneal motor amplitude as an independent predictor of accelerated renal decline and to explore potential effect modifications, prespecified subgroup analyses were performed. The cohort was stratified across clinically relevant baseline parameters, including gender, age (< 60 vs. ≥ 60 years), BMI (< 28.0 vs. ≥ 28.0 kg/m^2^), diabetes duration (< 10 vs. ≥ 10 years), HbA1c (< 8.0% vs. ≥ 8.0%), DPN, DKD, diabetic retinopathy (DR), hypertension, and the use of SGLT2 inhibitors. Within each stratum, the fully adjusted multivariable logistic regression model was reapplied to estimate the stratum‐specific ORs and 95% CIs. Formal interaction testing was conducted by introducing multiplicative interaction terms (e.g., peroneal motor amplitude × subgroup variable) into the main models to ascertain whether the prognostic capacity of the large‐fiber phenotype varied significantly across these diverse clinical profiles.

#### 2.5.5 Model Performance and Reclassification

To evaluate incremental prognostic utility, we first established a clinical base model comprising standard renal, metabolic, and therapeutic variables: baseline eGFR, serum albumin, platelet count, HbA1c, and SGLT2i use. The incremental prognostic value of incorporating neurofunctional phenotypes into the traditional clinical base model was evaluated. While discrimination was assessed using the area under the receiver operating characteristic curve (AUC), the continuous net reclassification improvement (NRI) and integrated discrimination improvement (IDI) were the primary metrics for evaluating clinical utility, which precisely quantify the correct reassignment of absolute risk probabilities for individual patients. Crucially, to rigorously address potential model overfitting and performance optimism, internal validation was performed using bootstrapping with 1000 resamples. This technique was applied to calculate the optimism‐corrected AUC and to generate robust 95% CIs for both the discriminative and reclassification metrics. Furthermore, model calibration was visually assessed using a calibration plot with LOESS smoothing and statistically evaluated via the Hosmer‐Lemeshow goodness‐of‐fit test to ensure agreement between predicted and observed risks.

All statistical analyses were performed using R software (version 4.4.2, R Foundation for Statistical Computing, Vienna, Austria). A two‐sided *p*‐value < 0.05 was considered statistically significant.

## 3 Results

### 3.1 Baseline Clinical Characteristics and Cross‐Sectional Association

Of the initial 1400 inpatients with Type 2 diabetes evaluated between 2020 and 2025, 694 eligible patients were included in the baseline cross‐sectional analysis after applying predefined exclusion criteria. Following the exclusion of patients lacking prospectively collected data, a final cohort of 234 patients was included in the longitudinal analysis (Figure 1).

**FIGURE 1:**
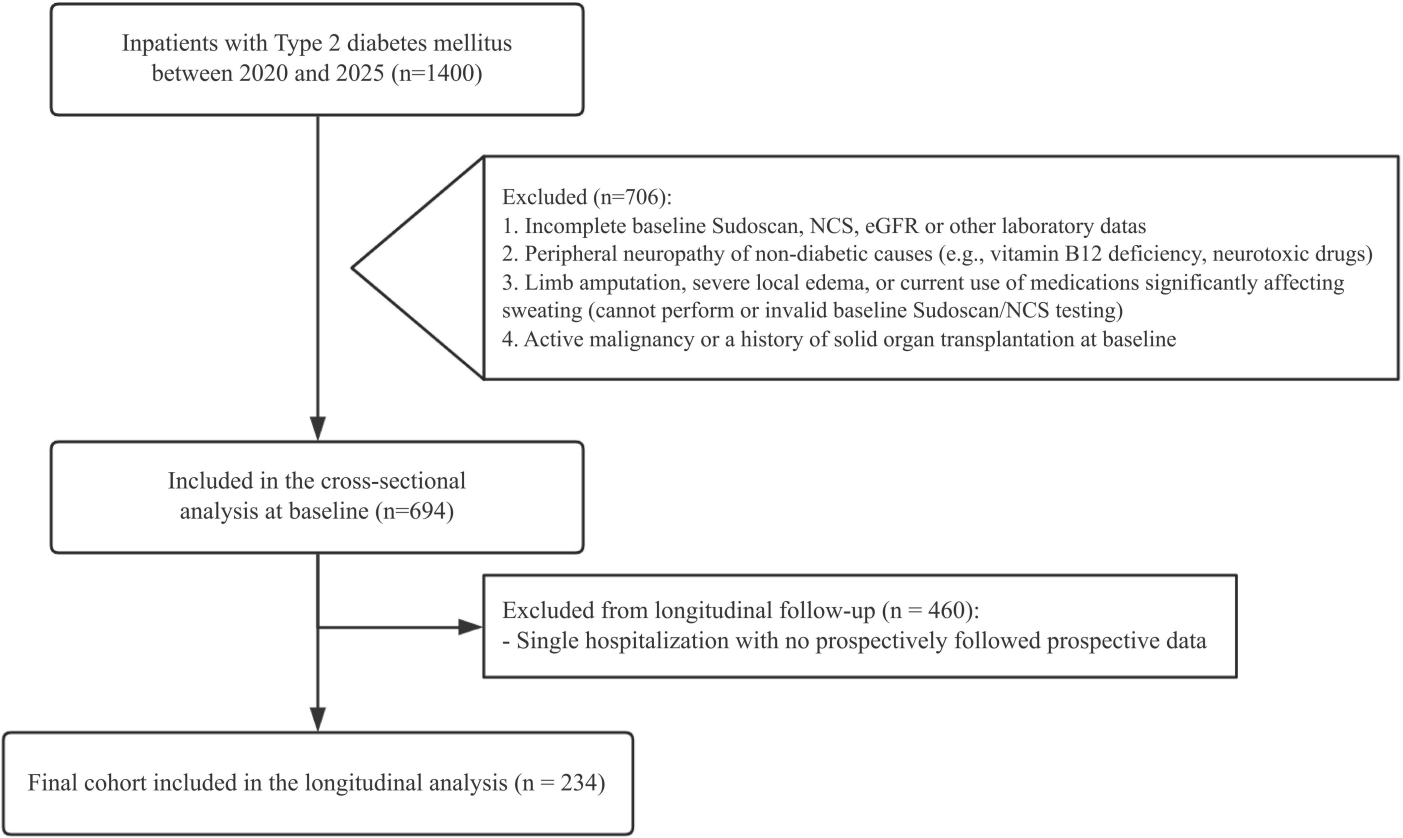
Flow diagram of study participant selection. The initial screening identified 1400 adult inpatients with Type 2 diabetes evaluated between 2020 and 2025. A total of 706 patients were systematically excluded based on predefined criteria, including incomplete baseline clinical or neurofunctional assessments, nondiabetic peripheral neuropathies, conditions precluding valid neurological testing, and severe concurrent systemic diseases. Consequently, 694 eligible patients were included in the baseline cross‐sectional analysis. Following the exclusion of 460 patients who lacked prospectively collected data, a final longitudinal cohort of 234 patients was established to assess the determinants of accelerated renal decline. eGFR, estimated glomerular filtration rate; NCS, nerve conduction study; SUDOSCAN, Sudomotor small‐fiber assessment.

Among the 694 patients in the cross‐sectional cohort, characterized by an overall median age of 64.50 years (IQR: 58.00–70.00) and a prolonged median diabetes duration of 16.00 years (IQR: 10.00–21.00), the participants were stratified into tertiles based on baseline ESCF (Table 1): T1 (most impaired, median 33.00 μS), T2 (66.00 μS), and T3 (most preserved, 80.00 μS).

**TABLE 1:** Baseline clinical and demographic characteristics of the study population stratified by ESCF.

Variables	Total (*n* = 694)	T1 (*n* = 232)	T2 (*n* = 231)	T3 (*n* = 231)	*p*
Male sex, *n* (%)	411 (59.22)	139 (59.91)	122 (52.81)	150 (64.94)	0.029
Age (years)	64.50 (58.00, 70.00)	66.00 (60.00, 72.00)	65.00 (58.00, 70.00)	61.00 (53.50, 68.00)	< 0.001
Diabetes duration (years)	16.00 (10.00, 21.00)	18.00 (11.00, 23.00)	16.00 (10.00, 22.00)	13.00 (8.00, 20.00)	< 0.001
BMI (kg/m^2^)	25.16 (23.10, 27.13)	25.18 (22.89, 26.84)	25.00 (23.24, 27.01)	25.25 (23.13, 27.41)	0.792
WC (cm)	94.00 (88.00, 101.00)	94.00 (89.00, 102.00)	92.00 (86.25, 98.00)	95.00 (88.00, 101.00)	0.286
Smoking (%)	293 (42.22)	102 (43.97)	90 (38.96)	101 (43.72)	0.470
Alcohol consumption (%)	279 (40.20)	94 (40.52)	80 (34.63)	105 (45.45)	0.060
SBP (mmHg)	131.00 (121.00, 141.00)	131.00 (121.00, 143.00)	132.00 (121.75, 141.00)	129.00 (121.00, 139.00)	0.265
DBP (mmHg)	78.00 (72.00, 87.00)	77.00 (72.00, 85.00)	79.50 (72.00, 87.00)	80.00 (74.00, 87.75)	0.045
HR (bpm)	74.00 (72.00, 78.00)	74.00 (71.50, 78.00)	74.00 (70.00, 78.00)	75.00 (72.00, 78.00)	0.280
HbA1c (%)	8.60 (7.38, 9.90)	9.10 (7.70, 10.40)	8.20 (7.20, 9.50)	8.60 (7.40, 9.70)	< 0.001
HbA1c (mmol/mol)	70.5 (57.2, 84.7)	76.0 (60.7, 90.2)	66.1 (55.2, 80.3)	70.5 (57.4, 82.5)	< 0.001
FBG (mmol/L)	7.20 (5.80, 9.20)	7.30 (5.70, 9.70)	7.00 (5.80, 8.85)	7.30 (6.00, 9.20)	0.329
FCP (pmol/L)	313.80 (179.18, 519.77)	306.85 (166.15, 555.42)	312.00 (183.50, 498.85)	330.00 (205.50, 505.00)	0.963
2hPBG (mmol/L)	11.60 (9.00, 14.80)	11.05 (8.67, 13.53)	12.35 (9.45, 15.50)	11.90 (9.00, 14.80)	0.103
2hCP (pmol/L)	917.60 (467.37, 1490.08)	998.50 (540.20, 1639.60)	982.50 (460.45, 1483.40)	849.30 (444.20, 1283.10)	0.238
WBC (*10^9^/L)	5.96 (4.96, 7.16)	6.14 (5.00, 7.22)	5.90 (4.92, 6.97)	5.99 (4.97, 7.08)	0.449
NEU (*10^9^/L)	3.43 (2.66, 4.33)	3.54 (2.74, 4.40)	3.36 (2.62, 4.22)	3.47 (2.70, 4.36)	0.352
LYM (*10^9^/L)	1.86 (1.45, 2.27)	1.82 (1.42, 2.24)	1.83 (1.52, 2.26)	1.91 (1.50, 2.30)	0.544
PLT (*10^9^/L)	205.00 (171.00, 242.00)	199.00 (166.00, 239.00)	205.00 (169.50, 240.00)	213.00 (175.00, 248.00)	0.104
TC (mmol/L)	3.99 (3.36, 4.66)	4.09 (3.45, 4.74)	3.86 (3.32, 4.51)	4.01 (3.30, 4.77)	0.339
TG (mmol/L)	1.26 (0.90, 1.98)	1.27 (0.94, 2.14)	1.23 (0.86, 1.75)	1.23 (0.90, 1.98)	0.232
LDL‐C (mmol/L)	2.24 (1.68, 2.82)	2.38 (1.80, 2.86)	2.15 (1.62, 2.72)	2.21 (1.68, 2.87)	0.140
HDL‐C (mmol/L)	1.09 (0.92, 1.32)	1.08 (0.88, 1.25)	1.13 (0.93, 1.34)	1.08 (0.92, 1.32)	0.223
ALT (g/L)	17.00 (13.00, 24.00)	17.00 (13.00, 24.00)	16.00 (13.00, 23.00)	17.00 (13.00, 25.00)	0.400
ALB (g/L)	40.00 (37.00, 42.00)	39.00 (37.00, 41.00)	40.00 (38.00, 42.00)	40.00 (38.00, 42.00)	< 0.001
UA (mmol/L)	341.00 (274.00, 401.00)	358.00 (282.00, 421.50)	326.00 (272.00, 393.50)	339.00 (273.00, 396.50)	0.076
Urea (mmol/L)	5.91 (4.82, 7.19)	6.17 (5.08, 7.61)	5.91 (4.78, 7.08)	5.70 (4.74, 6.83)	0.002
eGFR (mL/min/1.73 m^2^)	85.32 (66.17, 95.83)	77.49 (59.49, 90.79)	85.17 (69.07, 96.00)	89.98 (75.60, 99.53)	< 0.001
UACR (mg/g)	14.50 (7.70, 39.23)	11.70 (7.12, 28.48)	14.30 (8.25, 39.40)	17.60 (8.80, 49.00)	0.011
VD3 (ng/mL)	16.00 (11.90, 21.60)	14.80 (11.00, 21.50)	15.85 (12.00, 20.77)	16.55 (12.35, 22.60)	0.242
TSH (IU/mL)	1.84 (1.18, 2.68)	1.94 (1.14, 2.58)	1.73 (1.19, 2.79)	1.78 (1.19, 2.66)	0.951
FT4 (ng/dL)	2.93 (2.69, 3.18)	2.91 (2.68, 3.12)	2.92 (2.65, 3.20)	2.95 (2.73, 3.21)	0.499
FT3 (pg/mL)	1.16 (1.05, 1.29)	1.15 (1.04, 1.30)	1.17 (1.06, 1.29)	1.16 (1.06, 1.27)	0.872
Complications					
DKD (%)	374 (53.89)	132 (56.90)	128 (55.41)	114 (49.35)	0.226
DR (%)	212 (30.55)	81 (34.91)	70 (30.30)	61 (26.41)	0.138
Hypertension (%)	448 (64.55)	166 (71.55)	147 (63.64)	135 (58.44)	0.012
CVD (%)	286 (41.21)	110 (47.41)	99 (42.86)	77 (33.33)	0.007
NAFLD (%)	222 (31.99)	71 (30.60)	76 (32.90)	75 (32.47)	0.853
Osteoporosis (%)	89 (12.82)	34 (14.66)	31 (13.42)	24 (10.39)	0.369
Medications					
Insulin (%)	470 (67.72)	173 (74.57)	153 (66.23)	144 (62.34)	0.016
Metformin (%)	485 (69.88)	156 (67.24)	159 (68.83)	170 (73.59)	0.301
SGLT2i (%)	267 (38.47)	90 (38.79)	93 (40.26)	84 (36.36)	0.685
GLP‐1RA (%)	2 (0.29)	1 (0.43)	1 (0.43)	0 (0.00)	1.000
DPP4i (%)	167 (24.06)	54 (23.28)	55 (23.81)	58 (25.11)	0.894
α‐Glucosidase inhibitors (%)	258 (37.18)	99 (42.67)	83 (35.93)	76 (32.90)	0.084
Sulfonylureas (%)	6 (0.86)	2 (0.86)	2 (0.87)	2 (0.87)	1.000
Pioglitazone (%)	45 (6.48)	15 (6.47)	15 (6.49)	15 (6.49)	1.000
Nerve and parameter					
Right peroneal motor distal latency (ms)	4.20 (3.70, 5.40)	4.20 (3.70, 5.20)	4.10 (3.60, 5.10)	4.50 (3.80, 6.05)	0.006
Right peroneal motor amplitude (μV)	2.90 (1.60, 3.70)	2.40 (1.10, 3.20)	3.00 (1.90, 3.70)	3.10 (2.20, 3.90)	< 0.001
Right peroneal motor conduction velocity (m/s)	42.20 (38.60, 45.20)	40.50 (36.30, 43.40)	43.10 (39.55, 46.05)	43.00 (40.35, 45.50)	< 0.001
Right peroneal sensory amplitude (μV)	2.80 (2.20, 3.60)	2.80 (2.20, 3.50)	2.80 (2.30, 3.50)	2.90 (2.30, 3.70)	0.517
Right peroneal sensory conduction velocity (m/s)	48.20 (45.40, 51.10)	47.10 (41.18, 50.30)	48.55 (46.12, 51.20)	49.10 (46.30, 51.50)	< 0.001
ESCF (μS)	66.00 (44.00, 77.00)	33.00 (21.75, 44.00)	66.00 (59.00, 69.00)	80.00 (77.00, 85.00)	< 0.001
Asymmetry ratio between feet (%)	5.00 (2.00, 11.00)	12.00 (6.00, 20.25)	5.00 (2.00, 10.00)	2.00 (1.00, 4.00)	< 0.001

*Note:* Participants were stratified into four groups based on their baseline neurofunctional assessments: both normal, ISD, ILFD, and combined dysfunction. Data are presented as medians (IQRs) or *n* (%). *p*‐Values reflect overall comparisons across the four groups using the Kruskal–Wallis H test for continuous variables and the *χ* ^2^ test (or Fisher's exact test, as appropriate) for categorical variables.

Abbreviations: 2hCP, 2‐h postprandial C‐peptide; 2hPBG, 2‐h postprandial blood glucose; ALT, alanine aminotransferase; BMI, body mass index; CVD, cardiovascular disease; DBP, diastolic blood pressure; DKD, diabetic kidney disease; DPN, diabetic peripheral neuropathy; DPP4i, dipeptidyl peptidase‐4 inhibitors; DR, diabetic retinopathy; eGFR, estimated glomerular filtration rate; FBG, fasting blood glucose; FCP, fasting C‐peptide; GLP‐1RA, glucagon‐like peptide‐1 receptor agonists; HbA1c, glycated hemoglobin; HDL‐C, high‐density lipoprotein cholesterol; HR, heart rate; ILFD, isolated large‐fiber dysfunction; IQR, interquartile range; ISD, isolated sudomotor dysfunction; LDL‐C, low‐density lipoprotein cholesterol; LYM, lymphocyte; NAFLD, nonalcoholic fatty liver disease; NEU, neutrophil; PLT, platelet; SBP, systolic blood pressure; SGLT2i, sodium‐glucose cotransporter‐2 inhibitors; TC, total cholesterol; TG, triglycerides; UA, uric acid; UACR, urinary albumin‐to‐creatinine ratio; VD3, vitamin D3; WBC, white blood cell.

Compared to T3, patients in T1 exhibited older age (66.00 vs. 61.00 years, *p* < 0.001), longer diabetes duration (18.00 vs. 13.00 years, *p* < 0.001), poorer glycemic control (HbA1c 9.10% vs. 8.60%, *p* < 0.001), and a higher prevalence of cardiovascular disease (47.41% vs. 33.33%, *p* = 0.007). Notably, baseline renal function demonstrated stepwise deterioration alongside small‐fiber decline, with the eGFR decreasing progressively from T3 to T1 (89.98 vs. 85.17 vs. 77.49 mL/min/1.73 m^2^, *p* < 0.001). Large‐fiber parameters also progressively worsened, as evidenced by a significant, stepwise reduction in the median right peroneal motor amplitude (3.10 vs. 3.00 vs. 2.40 μV, *p* < 0.001).

The cross‐sectional relationship between the sudomotor small‐fiber function and baseline eGFR was assessed using multivariable linear regression (Supplemental Table 1). The baseline ESCF demonstrated a positive, independent correlation with the baseline eGFR across all sequential models. Accounting for demographics, traditional metabolic risk factors, and large‐fiber amplitude, this correlation remained significant (*β* = 0.18, 95% CI: 0.10–0.26, *p* < 0.001).

### 3.2 Independent Predictors of Accelerated Renal Decline

Among the 234 patients who completed the comprehensive longitudinal follow‐up, the core characteristics were highly comparable to those of the nonresponders across clinical, metabolic, neurofunctional parameters, and the prevalence of diabetic complications (Supplemental Table 2). To identify independent predictors of accelerated renal decline (> 3 mL/min/1.73 m^2^/year), a LASSO penalized regression was initially employed for variable selection. The LASSO algorithm retained serum albumin, platelet count, SGLT2i use, and right peroneal motor amplitude as the optimal subset of predictors (Supplemental Figure 1).

In Model 1 (the LASSO‐derived model, incorporating the aforementioned variables alongside baseline eGFR and ESCF), a higher baseline right peroneal motor amplitude independently predicted a lower risk of accelerated renal function decline (OR: 0.71, 95% CI: 0.55–0.90, *p* = 0.006) (Table 2). Additional significant predictors in this model included baseline eGFR, serum albumin, platelet count, and baseline SGLT2i use. Conversely, baseline ESCF was not significantly associated with the outcome (OR: 1.00, 95% CI: 0.99–1.02, *p* = 0.712).

**TABLE 2:** Multivariable logistic regression models evaluating independent predictors of rapid renal function decline.

Variables	Model 1: LASSO‐derived model		Model 2: Fully adjusted model	
	OR (95% CI)	*p*	OR (95% CI)	*p*
Metabolic and renal baseline				
Baseline eGFR	1.02 (1.00 ~ 1.03)	0.045	1.02 (1.00 ~ 1.04)	0.021
Serum albumin	0.85 (0.77 ~ 0.94)	0.002	0.86 (0.77 ~ 0.96)	0.008
Platelet count (per 50 × 10^9^/L)	0.69 (0.51 ~ 0.91)	0.011	0.72 (0.52 ~ 0.98)	0.044
Intervention				
SGLT2i Use (No vs. Yes)	2.15 (1.14 ~ 4.11)	0.019	2.02 (1.02 ~ 4.03)	0.043
Neurofunctional phenotypes				
ESCF	1.00 (0.99 ~ 1.02)	0.712	1.00 (0.99 ~ 1.02)	0.667
Peroneal motor amplitude	0.71 (0.55 ~ 0.90)	0.006	0.74 (0.56 ~ 0.95)	0.021
Traditional clinical confounders				
Age			1.01 (0.98 ~ 1.05)	0.529
Sex (Male vs. Female)			1.88 (0.92 ~ 3.93)	0.085
Diabetes duration			1.03 (0.99 ~ 1.07)	0.201
HbA1c			1.01 (0.83 ~ 1.21)	0.950
SBP			1.00 (0.98 ~ 1.03)	0.652
DR (Yes vs. No)			0.71 (0.34 ~ 1.46)	0.364

*Note:* Rapid renal function decline was defined a priori as an annualized eGFR decrease of > 3 mL/min/1.73 m^2^. *Model 1* represents the predictive model incorporating covariates optimally selected via the least absolute shrinkage and selection operator (LASSO) penalized regression, deliberately forced with the pre‐specified core exposures (baseline eGFR and ESCF). *Model 2* represents the fully adjusted multivariable logistic regression model incorporating traditional demographic, clinical, and metabolic confounders.

Abbreviations: CI, confidence interval; DR, diabetic retinopathy; eGFR, estimated glomerular filtration rate; ESCF, electrochemical skin conductance of feet; HbA1c, glycated hemoglobin; OR, odds ratio; SBP, systolic blood pressure; SGLT2i, sodium‐glucose cotransporter‐2 inhibitors.

In Model 2, the independent predictive value of right peroneal motor amplitude remained statistically significant (OR: 0.74, 95% CI: 0.56–0.95, *p* = 0.021), which additionally incorporated traditional clinical confounders, including age, sex, diabetes duration, HbA1c, systolic blood pressure, and diabetic retinopathy (Table 2). Baseline ESCF persistently lacked any significant association with the longitudinal outcome (OR: 1.00, 95% CI: 0.99–1.02, *p* = 0.667).

To rigorously validate the robustness of large‐fiber integrity as a predictor, a sensitivity analysis was performed using the more conservative KDIGO threshold for rapid disease progression (> 5 mL/min/1.73 m^2^/year) (Supplemental Table 3). Notably, a higher baseline right peroneal motor amplitude remained a significant independent protective factor against extreme renal decline (OR: 0.76; 95% CI: 0.61–0.95; *p* = 0.016). The baseline ESCF remained entirely nonsignificant (OR: 1.00, 95% CI: 0.98–1.02, *p* = 0.843).

### 3.3 Attenuation of Sudomotor Prediction by Baseline Renal Function

The association between baseline ESCF and follow‐up eGFR was further evaluated using sequential multivariable linear regression (Table 3), with follow‐up eGFR as the dependent variable. Baseline ESCF exhibited a significant positive association with follow‐up eGFR in the crude model (*β* = 0.28, 95% CI: 0.14–0.42, *p* < 0.001) and remained significant after adjusting for demographic and traditional metabolic risk factors (Model 2: *β* = 0.18, 95% CI: 0.04–0.31, *p* = 0.009). However, upon the addition of baseline eGFR to the fully adjusted model, the association between ESCF and follow‐up eGFR was substantially attenuated and lost statistical significance (Model 4: *β* = 0.05, 95% CI: −0.03 to 0.14, *p =* 0.210). This pronounced attenuation confirms that the apparent predictive value of sudomotor function for future renal trajectories is heavily confounded by the baseline renal foundation.

**TABLE 3:** Longitudinal multivariable linear regression models evaluating the association between baseline sudomotor small‐fiber function and follow‐up eGFR.

Variables	Model 1		Model 2		Model 3		Model 4	
	*β* (95% CI)	*p*	*β* (95% CI)	*p*	*β* (95% CI)	*p*	*β* (95% CI)	*p*
Baseline ESCF	0.28 (0.14 ~ 0.42)	< 0.001	0.18 (0.04 ~ 0.31)	0.009	0.08 (−0.07 ~ 0.22)	0.298	0.05 (−0.03 ~ 0.14)	0.210

*Note:* Unstandardized *β* coefficients and 95% CIs from linear regression models predicting follow‐up eGFR based on baseline ESCF within the longitudinal sub‐cohort (*n* = 234). *Model 1*: Unadjusted crude model. *Model 2*: Adjusted for age, sex, smoking status, alcohol consumption, diabetes duration, BMI, SBP, HbA1c, serum albumin, platelet count, and the utilization of SGLT2i. *Model 3*: Adjusted for Model 2 covariates plus baseline right peroneal motor nerve amplitude. *Model 4*: Adjusted for Model 2 covariates plus baseline eGFR.

Abbreviations: BMI, body mass index; CI, confidence interval; eGFR, estimated glomerular filtration rate; ESCF, electrochemical skin conductance of feet; HbA1c, glycated hemoglobin; SBP, systolic blood pressure; SGLT2i, sodium‐glucose cotransporter‐2 inhibitors.

### 3.4 Subgroup Analyses for Robustness and Interaction

To explore the consistency of the large motor nerve phenotype's prognostic value across clinical strata, prespecified subgroup analyses were conducted. Subgroup‐specific adjusted ORs, 95% CIs, and formal interaction *p*‐values are comprehensively detailed in Figure 2. The inverse prognostic association of a higher baseline peroneal motor amplitude against accelerated renal decline was consistently maintained across the majority of subgroups, including age, BMI, diabetes duration, HbA1c control, and SGLT2i utilization (all *p* for interaction > 0.05).

**FIGURE 2:**
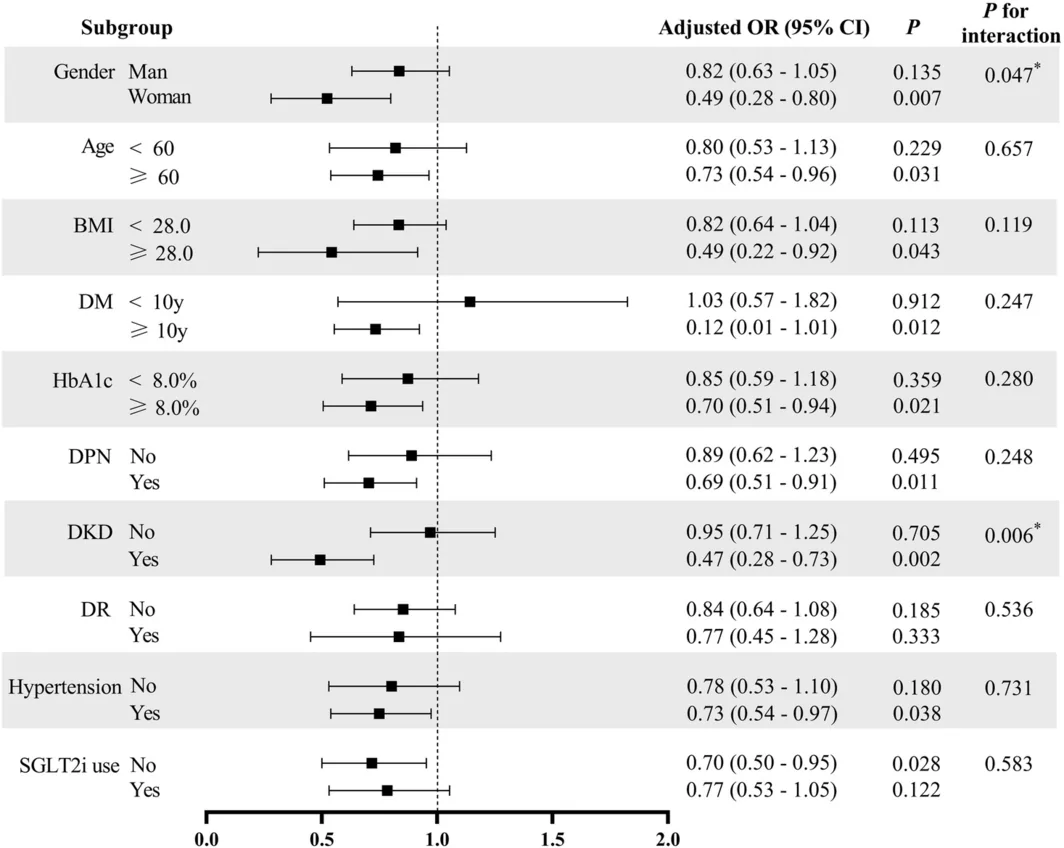
Subgroup analyses of the association between baseline peroneal motor amplitude and the risk of accelerated renal decline. Forest plot illustrating the adjusted ORs and 95% CI for accelerated renal decline (defined as an annualized eGFR loss of > 3 mL/min/1.73 m^2^/year) associated with baseline right peroneal motor amplitude across diverse clinical strata. The vertical dashed line represents an OR of 1.0 (indicating no effect). To calculate the adjusted ORs, the multivariable logistic regression model was reapplied within each subgroup, consistently adjusted for baseline eGFR, serum albumin, and platelet count. The *p*‐value denotes the statistical significance of the peroneal motor amplitude within each individual stratum. The *p* for interaction evaluates whether the prognostic capacity of the large‐fiber phenotype is significantly modified by the subgroup variables. An asterisk (*) highlights a statistically significant interaction effect (*p* for interaction < 0.05), which was specifically observed for baseline gender and DKD status. BMI, body mass index; CI, confidence interval; DKD, diabetic kidney disease; DM, diabetes mellitus; DPN, diabetic peripheral neuropathy; DR, diabetic retinopathy; eGFR, estimated glomerular filtration rate; HbA1c, glycated hemoglobin; OR, odds ratio; SGLT2i, sodium‐glucose cotransporter‐2 inhibitor.

Notably, formal interaction testing revealed significant effect modifications by baseline DKD status (*p* for interaction = 0.006) and gender (*p* for interaction = 0.047). The prognostic capacity of peroneal motor amplitude was significant in patients with pre‐existing DKD (OR: 0.47, 95% CI: 0.28–0.73, *p* = 0.002), whereas it demonstrated no significant association in those without baseline DKD (OR: 0.95, 95% CI: 0.71–1.25, *p* = 0.705). Additionally, this predictive neuro‐renal association appeared more pronounced in female patients (OR: 0.49, 95% CI: 0.28–0.80, *p* = 0.007) compared to male patients (OR: 0.82, 95% CI: 0.63–1.05, *p* = 0.135).

### 3.5 Incremental Predictive Value and Risk Reclassification

The integration of the neurofunctional phenotype into the traditional clinical base model provided a modest improvement in apparent risk discrimination, with the AUC increasing from 0.666 (95% CI: 0.593–0.742) to 0.705 (95% CI: 0.635–0.770) (Figure 3A). To address the potential for model overfitting, internal validation via bootstrapping with 1000 resamples was performed. The estimated average optimism bias was remarkably low at 0.042, resulting in an optimism‐corrected AUC of 0.664 for the combined model. Model calibration was excellent, as visually confirmed by the calibration plot (Supplemental Figure 2) and statistically validated by the Hosmer‐Lemeshow test (*p* = 0.445). DCA confirmed the practical utility of this neuro‐renal approach, demonstrating that the combined model provided a superior net clinical benefit across a broad range of threshold probabilities compared with the clinical base model alone (Figure 3B).

**FIGURE 3:**
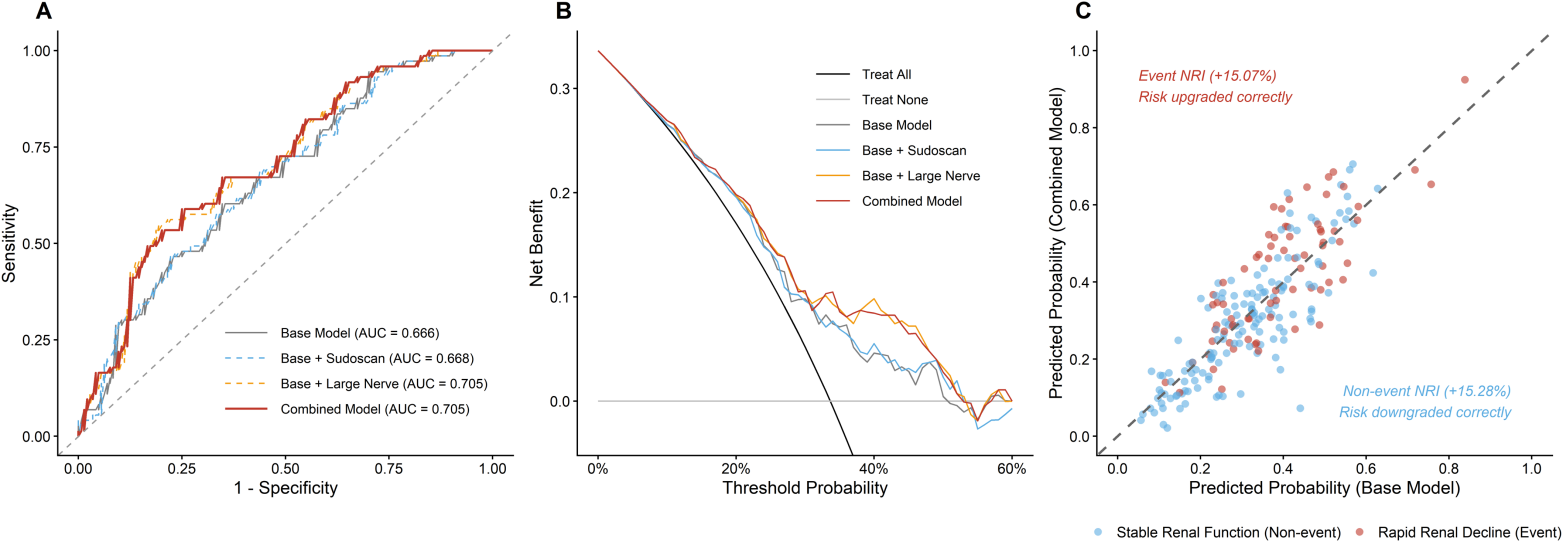
Incremental prognostic value and clinical utility of integrating neurofunctional parameters for predicting accelerated renal decline. (A) ROC curves demonstrating the discriminative performance of the distinct predictive models. The addition of large‐fiber parameters substantially improved the predictive capacity, increasing the AUC from 0.666 (Base Model) to 0.705 (Base + Large Nerve; Combined Model). The isolated addition of sudomotor parameters yielded negligible improvement (Base + Sudoscan, AUC = 0.668). (B) DCA evaluating the net clinical benefit across a continuum of threshold probabilities. The combined model and the large‐nerve‐integrated model demonstrated superior net benefit compared to the base clinical model and the default “treat‐all” or “treat‐none” strategies. (C) Continuous NRI scatter plot illustrating individual‐level risk recalibration between the base and combined models. The integrated approach successfully reclassified risk trajectories, achieving an event NRI of +15.07% (risk upgraded correctly for patients experiencing rapid renal decline) and a nonevent NRI of +15.28% (risk downgraded correctly for patients with stable renal function). AUC, area under the curve; DCA, decision curve analysis; NRI, net reclassification improvement; ROC, receiver operating characteristic.

Beyond traditional discrimination and cohort‐level clinical benefit, the combined model achieved a clinically relevant continuous NRI of 30.35% (95% CI: 3.80%–67.98%). As visually demonstrated by the individual‐level reclassification scatter plot (Figure 3C), this significant risk recalibration was driven: the combined model correctly assigned higher risk probabilities to patients who subsequently experienced accelerated renal decline (event NRI: +15.07%), while accurately downgrading the risk probabilities of those who remained stable (nonevent NRI: +15.28%). This superior recalibration accuracy was further corroborated by an IDI of 0.0365 (95% CI: 0.006–0.106, *p* = 0.079).

## 4 Discussion

In this real‐world, longitudinal cohort study of patients with Type 2 diabetes, we identified a distinct prognostic dissociation within the neuro‐renal axis. Although sudomotor small‐fiber impairment accurately mirrored baseline eGFR cross‐sectionally, it failed to independently predict accelerated renal decline, as its longitudinal prognostic value was fundamentally confounded by the baseline renal foundation. Conversely, the peroneal motor amplitude emerged as a robust and independent predictor of future accelerated renal decline. Notably, integrating nerve phenotype into a traditional clinical base model yielded a substantial continuous NRI of 30.35%, providing a clinically meaningful, individualized risk recalibration that extends significantly beyond standard metabolic risk factors.

Our cross‐sectional findings confirmed that sudomotor small‐fiber dysfunction is significantly correlated with concurrent renal impairment [7, 13, 14]. However, the longitudinal phase of our cohort revealed that the prognostic value of ESCF for accelerated renal function decline was largely dependent on baseline eGFR. Sharing a highly susceptible microvascular network, the neuro‐sudomotor and renal glomerular systems sustain parallel damage; thus, baseline sudomotor dysfunction has already fully reflected the preexisting renal microvascular ischemic burden. Consequently, rather than acting as an independent pathogenic driver of ongoing nephron loss, sudomotor small‐fiber dysfunction serves primarily as a high‐fidelity cross‐sectional mirror of concurrent structural renal damage, lacking the capacity to yield incremental prognostic utility for future renal trajectories beyond the established baseline foundation.

In advanced, multimorbid diabetic populations, disease progression is predominantly driven by established downstream structural organ damage rather than transient metabolic fluctuations. Consequently, traditional clinical parameters, including HbA1c and duration of diabetes, were excluded from the optimal predictive subset during LASSO penalized regression within this specific cohort. Serum albumin, platelet count, SGLT2i use, and right peroneal motor amplitude were retained as the independent predictors of future renal trajectories. Serum albumin and platelet counts likely serve as composite indicators of chronic systemic inflammation, endothelial microthrombosis, and the ongoing burden of protein‐losing nephropathy [15]. Concurrently, the retention of SGLT2i use highlights the profound disease‐modifying efficacy of modern renoprotective therapies, which fundamentally alter long‐term renal trajectories independent of historical glycemic control [16]. Ultimately, the selection of the peroneal motor amplitude suggests that macro‐structural axonal degeneration acts as a comprehensive surrogate marker for cumulative systemic damage. This axonal loss reflects the shared vulnerability of the peripheral nervous system and the kidneys to generalized microvascular disease, overall diabetes severity, and broader systemic factors such as frailty and nutritional status [5].

Subgroup analyses further explored the consistency of the large‐fiber phenotype's prognostic value. Its predictive value remained consistent regardless of baseline HbA1c or SGLT2i use, demonstrating independence from transient metabolic fluctuations and modern disease‐modifying therapies. Notably, a significant interaction with baseline DKD status emerged: the prognostic association of large nerve degeneration was significant primarily in patients with established DKD, aligning with the concept that it acts as a surrogate for advanced end‐organ damage. Similarly, this neuro‐renal association was more pronounced in women, potentially reflecting increased microvascular vulnerability linked to the postmenopausal loss of estrogen‐mediated protection. Nevertheless, these subgroup observations remain strictly exploratory and warrant dedicated mechanistic investigation.

From a prognostic perspective, incorporating the neurofunctional phenotype into standard clinical models provides a refined, rather than merely additive, value for individualized risk assessment. While the improvement in overall discriminative capacity was modest (AUC increasing from 0.666 to 0.705), the combined model achieved a clinically relevant continuous NRI of 30.35%. This precise recalibration indicates that the model successfully identified a significant proportion of patients destined for accelerated renal decline who would have otherwise been erroneously classified as low‐risk by conventional demographic and metabolic parameters (event NRI: +15.07%). Correspondingly, it accurately downgraded the predicted risk for stable patients (nonevent NRI: +15.28%). Furthermore, DCA confirmed that this integrated model yields a superior net clinical benefit across a broad range of threshold probabilities, directly mitigating the potential for over‐medicalization. In real‐world practice, capturing this subgroup of “hidden high‐risk” patients provides a critical window for targeted intervention. Proactive clinical decisions—such as the early initiation of SGLT2 inhibitors and the implementation of stringent multidisciplinary follow‐up—could be pivotal in arresting further progression toward end‐stage renal disease.

Several limitations should be considered when interpreting our findings. First, the lack of systematic tracking for out‐of‐network outcomes (e.g., ESRD or mortality) means that the potential for informative censoring cannot be definitively excluded. Second, although SUDOSCAN is a validated and highly reproducible tool, the lack of confirmation via gold‐standard modalities (e.g., skin biopsies) precludes the definitive anatomical characterization of small‐fiber structural damage. Third, our longitudinal outcome assessment relied on a two‐point calculation of annualized eGFR decline, which inherently assumes linear progression and may not capture acute intra‐individual fluctuations or nonlinear trajectories. Fourth, despite multivariable adjustments, we cannot exclude residual confounding from unmeasured systemic factors including generalized frailty and nutritional status, which may contemporaneously drive both axonal loss and nephropathy. Fifth, without multiple testing corrections, subgroup findings remain strictly exploratory, as subdividing the cohort reduces the event‐per‐variable ratio and statistical stability. Finally, given the observational design, modest overall sample size, and limited number of outcome events from a single center, our predictive modeling currently lacks external validation; thus, reclassification metrics (e.g., NRI) remain exploratory pending large‐scale multicenter confirmation.

In conclusion, the results of this longitudinal cohort study revealed a distinct prognostic dissociation within the diabetic neuro‐renal axis. Sudomotor small‐fiber dysfunction serves primarily as a high‐fidelity cross‐sectional mirror of the preexisting microvascular complications. Conversely, reduced peroneal motor amplitude serves as an associated marker of future accelerated renal decline that requires external validation. For patients with advanced Type 2 diabetes, incorporating this neurofunctional phenotype into traditional clinical models yields clinically relevant individualized risk reclassification, potentially aiding in the identification of high‐risk individuals. Ultimately, these findings advocate for a critical paradigm shift in late‐stage risk stratification; transitioning the prognostic focus from transient metabolic fluctuations toward the objective assessment of irreversible, macro‐structural end‐organ damage.

## Funding

This study was supported by the National Science and Technology Major Project for Noncommunicable Chronic Diseases (Grant Nos. 2024ZD0531900 and 2024ZD0531905), the National Natural Science Foundation of China (Grant No. 82270881), and the Capital's Funds for Health Improvement and Research (Grant No. 2024‐1‐4053).

## Ethics Statement

The study was conducted in accordance with the Declaration of Helsinki and was approved by the Institutional Review Board of Beijing Hospital (Approval Number 2024BJYYEC‐KY083‐02). A waiver of informed consent was granted due to the retrospective and anonymized nature of the clinical data.

## Conflicts of Interest

The authors declare no conflicts of interest.

## Supporting information


**Supplemental Figure 1:** Variable selection for accelerated renal decline using the LASSO penalized regression algorithm. (A). LASSO coefficient profiles of the candidate clinical, metabolic, and neurofunctional variables. The penalized regression algorithm applies a tuning parameter (*λ*) that shrinks the coefficients of less contributory prognostic variables toward zero. The *x*‐axis represents the natural logarithm of *λ* (log(*λ*)), and the *y*‐axis displays the corresponding coefficient values for each candidate variable.
(B). Selection of the optimal penalization parameter (*λ*) via 10‐fold cross‐validation. The binomial deviance (*y*‐axis) is plotted against log(*λ*) (bottom *x*‐axis). The numbers along the top *x*‐axis (ranging from 90 to 0) indicate the number of nonzero coefficients retained in the model at each corresponding *λ* value. The left and right vertical dashed lines denote the optimal values of *λ* derived from the minimum deviance criteria *λ*_min_ and the one standard error of the minimum criteria *λ*_1se_, respectively. Based on the optimal threshold, the algorithm successfully eliminated redundant parameters and retained the definitive predictive subset: serum albumin, platelet count, SGLT2i use, and right peroneal motor amplitude. LASSO, least absolute shrinkage and selection operator.

**Supplemental Figure 2:** Calibration plot of the combined predictive model for accelerated renal decline. The dashed gray line represents ideal perfect calibration, while the solid red line represents the apparent calibration accuracy fitted using LOESS smoothing, with the shaded area indicating the 95% confidence interval. The Hosmer‐Lemeshow goodness‐of‐fit test yielded a *p* value of 0.445, indicating excellent model calibration.

**Supplemental Table 1:** Cross‐sectional multivariable linear regression models evaluating the association between sudomotor small‐fiber function and baseline eGFR.
**Supplemental Table 2:** Comparison of baseline clinical and demographic characteristics between patients with and without longitudinal follow‐up.
**Supplemental Table 3:** Sensitivity analysis: Multivariable logistic regression model predicting accelerated renal decline defined by a stricter threshold (> 5 mL/min/1.73 m^2^/year).

## Data Availability

The data that support the findings of this study are available from the corresponding author upon reasonable request.
