# Supplementary material for: Prognostic Value of Sudomotor Small‐Fiber Function and Large Nerves for Accelerated Renal Decline in Type 2 Diabetes: A Real‐World Cohort Study
Source: J Diabetes. 2026 Oct 19;18(10):e70286. doi: 10.1111/1753-0407.70286 (PMC13680109; doi:10.1111/1753-0407.70286)
Supplement: Supplementary file 1 [file JDB-18-e70286-s002.docx]

Supplemental Table 1. Cross-sectional multivariable linear regression models evaluating the association between sudomotor small-fibre function and baseline eGFR

| Variables | Model1 | |  | Model2 | |  | Model3 | |  | Model4 | |
| --- | --- | --- | --- | --- | --- | --- | --- | --- | --- | --- | --- |
|  | β (95%CI) | *P* |  | β (95%CI) | *P* |  | β (95%CI) | *P* |  | β (95%CI) | *P* |
| ESCF | 0.24 (0.17 ~ 0.31) | <0.001 |  | 0.18 (0.12 ~ 0.24) | <0.001 |  | 0.19 (0.12 ~ 0.26) | <0.001 |  | 0.17 (0.10 ~ 0.25) | <0.001 |

Unstandardised β coefficients and 95% CIs from linear regression models predicting baseline eGFR based on the ESCF.

Model 1: Unadjusted crude model.

Model 2: Adjusted for age, sex, smoking status, alcohol consumption, diabetes duration, BMI, and SBP.

Model 3: Adjusted for Model 2 covariates plus HbA1c, serum albumin, platelet count, and the utilisation of SGLT2i.

Model 4: Adjusted for Model 3 covariates plus right peroneal motor nerve amplitude.

Abbreviations: BMI: Body mass index, CI: Confidence interval, eGFR: Estimated glomerular filtration rate, ESCF: Electrochemical skin conductance of feet, HbA1c: Glycated haemoglobin, SBP: Systolic blood pressure, SGLT2i: Sodium-glucose cotransporter-2 inhibitors.

Supplemental Table 2. Comparison of baseline clinical and demographic characteristics between patients with and without longitudinal follow-up

| Variables | No follow-up (n=460) | Follow-up (n=234) | *P* |
| --- | --- | --- | --- |
| Male sex, n (%) | 283 (60.7%) | 128 (56.1%) | 0.283 |
| Age (years) | 65.00 (58.00, 71.00) | 64.00 (57.00, 70.00) | 0.609 |
| Diabetes Duration (years) | 16.00 (10.00, 22.00) | 16.00 (10.00, 21.00) | 0.578 |
| BMI (kg/m^2^) | 25.22 (23.11, 27.26) | 24.95 (23.03, 26.89) | 0.465 |
| WC (cm) | 93.75 (87.00, 101.00) | 94.00 (88.00, 101.00) | 0.559 |
| Smoking (%) | 199 (42.7%) | 94 (41.2%) | 0.773 |
| Alcohol consumption (%) | 193 (41.4%) | 86 (37.7%) | 0.395 |
| SBP (mmHg) | 131.00 (121.75, 141.00) | 128.50 (121.00, 140.75) | 0.350 |
| DBP (mmHg) | 78.50 (72.00, 87.00) | 78.00 (71.25, 86.00) | 0.143 |
| HR (bpm) | 74.00 (70.00, 78.00) | 74.00 (72.00, 78.00) | 0.958 |
| HbA1c (%) | 8.70 (7.50, 9.90) | 8.30 (7.30, 9.90) | 0.222 |
| HbA1c (mmol/mol) | 71.6 (58.5, 84.7) | 67.2 (56.3, 84.7) | 0.222 |
| FBG (mmol/L) | 7.20 (5.80, 9.17) | 7.10 (5.90, 9.40) | 0.985 |
| FCP (pmol/L) | 335.00 (183.30, 570.10) | 289.20 (177.50, 467.20) | 0.031 |
| 2hPBG (mmol/L) | 11.60 (8.90, 14.30) | 11.95 (9.00, 15.50) | 0.280 |
| 2hCP (pmol/L) | 915.50 (487.45, 1448.25) | 924.30 (459.30, 1543.30) | 0.948 |
| WBC (*10^9^/L) | 5.93 (4.99, 7.20) | 6.06 (4.91, 7.05) | 0.464 |
| NEU (*10^9^/L) | 3.47 (2.72, 4.38) | 3.37 (2.52, 4.09) | 0.104 |
| LYM (*10^9^/L) | 1.85 (1.45, 2.26) | 1.88 (1.49, 2.29) | 0.569 |
| PLT (*10^9^/L) | 205.00 (171.00, 241.00) | 205.00 (170.50, 244.00) | 0.854 |
| TC (mmol/L) | 3.95 (3.35, 4.60) | 4.10 (3.40, 4.95) | 0.075 |
| TG (mmol/L) | 1.29 (0.92, 2.02) | 1.18 (0.85, 1.92) | 0.189 |
| LDL-C (mmol/L) | 2.20 (1.68, 2.74) | 2.29 (1.66, 3.01) | 0.213 |
| HDL-C (mmol/L) | 1.09 (0.92, 1.30) | 1.10 (0.91, 1.34) | 0.597 |
| ALT (g/L) | 17.00 (13.00, 24.00) | 17.00 (12.75, 23.25) | 0.471 |
| UA (mmol/L) | 342.00 (280.00, 406.00) | 337.00 (266.00, 396.00) | 0.273 |
| Urea (mmol/L) | 5.91 (4.83, 7.15) | 5.90 (4.81, 7.22) | 0.657 |
| eGFR (mL/min/1.73m²) | 84.14 (65.74, 95.30) | 86.91 (69.37, 97.13) | 0.274 |
| UACR (mg/g) | 14.50 (7.75, 38.70) | 13.80 (7.70, 39.10) | 0.866 |
| VD3 (ng/mL) | 16.25 (12.00, 21.58) | 15.90 (11.50, 21.60) | 0.867 |
| TSH (IU/mL) | 1.77 (1.18, 2.64) | 1.94 (1.16, 2.73) | 0.375 |
| FT4 (ng/dL) | 1.16 (1.06, 1.28) | 1.16 (1.03, 1.29) | 0.716 |
| FT3 (pg/mL) | 2.91 (2.67, 3.18) | 2.96 (2.74, 3.18) | 0.290 |
| Complications | | | |
| DKD (%) | 250 (53.6%) | 124 (54.4%) | 0.919 |
| DR (%) | 138 (29.6%) | 57 (25.0%) | 0.406 |
| Hypertension (%) | 303 (65.0%) | 145 (63.6%) | 0.776 |
| CVD (%) | 189 (40.6%) | 97 (42.5%) | 0.677 |
| NAFLD (%) | 151 (32.4%) | 71 (31.1%) | 0.804 |
| Osteoporosis (%) | 60 (12.9%) | 29 (12.7%) | 1.000 |
| Medications | | | |
| Insulin (%) | 322 (69.1%) | 148 (64.9%) | 0.307 |
| Metformin (%) | 337 (72.3%) | 148 (64.9%) | 0.056 |
| SGLT2i (%) | 191 (41.0%) | 76 (33.3%) | 0.062 |
| GLP-1RA (%) | 1 (0.2%) | 1 (0.4%) | 0.549 |
| DPP4i (%) | 103 (22.1%) | 64 (28.1%) | 0.103 |
| α-glucosidase inhibitors (%) | 179 (38.4%) | 79 (34.6%) | 0.379 |
| Sulfonylureas (%) | 6 (1.3%) | 0 (0.0%) | 0.185 |
| Pioglitazone (%) | 41 (8.8%) | 4 (1.8%) | < 0.001 |
| Nerve and Parameter | | | |
| Right peroneal motor distal latency (ms) | 4.20 (3.60, 5.10) | 4.40 (3.80, 6.80) | 0.003 |
| Right peroneal motor amplitude (μV) | 2.80 (1.60, 3.70) | 2.90 (1.90, 3.60) | 0.620 |
| Right peroneal motor conduction velocity (m/s) | 42.00 (38.20, 45.00) | 42.55 (39.55, 45.50) | 0.059 |
| Right peroneal sensory amplitude (μV) | 2.80 (2.30, 3.50) | 2.80 (2.20, 3.70) | 0.941 |
| Right peroneal sensory conduction velocity (m/s) | 47.90 (44.88, 50.90) | 48.70 (46.10, 51.60) | 0.046 |
| ESCF (µS) | 65.50 (42.00, 77.00) | 66.00 (47.75, 77.00) | 0.304 |
| Asymmetry ratio between feet (%) | 5.00 (2.00, 11.00) | 5.00 (2.00, 10.00) | 0.901 |

Comparison of the baseline profiles of patients who completed the longitudinal follow-up (n = 287) against those who were excluded due to a lack of prospective follow-up data (n = 438). Data are presented as medians (IQRs) for continuous variables, and as counts (%) for categorical variables. Differences between the two cohorts were evaluated using the Mann–Whitney U test for continuous variables and the χ² test (or Fisher’s exact test, as appropriate) for categorical variables. Statistically significant variations between the cohorts were observed in select baseline metrics, including HbA1c, FCP, TG and the utilization of specific medications (sulfonylureas and pioglitazone).

Abbreviations: 2hCP: 2-hour postprandial C-peptide, 2hPBG: 2-hour postprandial blood glucose, ALT: Alanine aminotransferase, BMI: Body mass index, CVD: Cardiovascular disease, DBP: Diastolic blood pressure, DKD: Diabetic kidney disease, DPN: Diabetic peripheral neuropathy, DPP4i: Dipeptidyl peptidase-4 inhibitors, DR: Diabetic retinopathy, eGFR: Estimated glomerular filtration rate, FBG: Fasting blood glucose, FCP: Fasting C-peptide, GLP-1RA: Glucagon-like peptide-1 receptor agonists, HbA1c: Glycated haemoglobin, HDL-C: High-density lipoprotein cholesterol, HR: Heart rate, ILFD: Isolated large-fibre dysfunction, IQR: Interquartile range, ISD: Isolated sudomotor dysfunction, LDL-C: Low-density lipoprotein cholesterol, LYM: Lymphocyte, NAFLD: Non-alcoholic fatty liver disease, NEU: Neutrophil, PLT: Platelet, SBP: Systolic blood pressure, SGLT2i: Sodium-glucose cotransporter-2 inhibitors, TC: Total cholesterol, TG: Triglycerides, UA: Uric acid, UACR: Urinary albumin-to-creatinine ratio, VD3: Vitamin D3, WBC: White blood cell.

Supplemental Table 3. Sensitivity analysis: Multivariable logistic regression model predicting accelerated renal decline defined by a stricter threshold (> 5 mL/min/1.73 m²/year)

| Variables | OR (95% CI) | *P* value |
| --- | --- | --- |
| **Metabolic & Renal Baseline** | |  |
| Baseline eGFR | 1.02 (1.00 ~ 1.04) | 0.034 |
| Serum Albumin | 0.86 (0.77 ~ 0.96) | 0.008 |
| Platelet Count (per 50 ×10^9^/L) | 0.70 (0.51 ~ 0.94) | 0.023 |
| **Intervention** |  |  |
| SGLT2i Use (No vs. Yes) | 1.62 (1.10 ~ 2.38) | 0.014 |
| **Neurofunctional Phenotypes** | |  |
| ESCF | 1.00 (0.98 ~ 1.02) | 0.843 |
| Peroneal Motor Amplitude | 0.76 (0.61 ~ 0.95) | 0.016 |

This sensitivity analysis applies a stricter a priori threshold for the primary outcome, defining accelerated renal function decline as an annualised eGFR decrease of > 5 mL/min/1.73 m². The multivariable logistic regression model incorporates covariates optimally selected via the least absolute shrinkage and selection operator (LASSO) penalised regression in the primary analysis. Pre-specified core exposures (baseline eGFR and ESCF) were forced into the model to ensure direct methodological comparability with the main findings.

Abbreviations: CI: Confidence interval, eGFR: Estimated glomerular filtration rate, ESCF: Electrochemical skin conductance of feet, OR: Odds ratio, SGLT2i: Sodium-glucose cotransporter-2 inhibitors.
